# Ambient-pressure endstation of the Versatile Soft X-ray (VerSoX) beamline at Diamond Light Source

**DOI:** 10.1107/S1600577520009157

**Published:** 2020-08-17

**Authors:** Georg Held, Federica Venturini, David C. Grinter, Pilar Ferrer, Rosa Arrigo, Liam Deacon, Wilson Quevedo Garzon, Kanak Roy, Alex Large, Christopher Stephens, Andrew Watts, Paul Larkin, Matthew Hand, Hongchang Wang, Linda Pratt, James J. Mudd, Thomas Richardson, Suren Patel, Michael Hillman, Stewart Scott

**Affiliations:** a Diamond Light Source Ltd, Oxfordshire, United Kingdom; b Department of Chemistry, University of Reading, Reading, United Kingdom; c School of Science, Engineering and Environment, University of Salford, Manchester, United Kingdom; d Helmholtz-Zentrum Berlin für Materialien und Energie, Berlin, Germany

**Keywords:** soft X-ray beamline, ambient-pressure XPS, X-ray absorption, catalysis, carbon contamination

## Abstract

The ambient-pressure endstation and branchline of the Versatile Soft X-ray (VerSoX) beamline B07 at Diamond Light Source serves a very diverse user community studying heterogeneous catalysts, pharmaceuticals and biomaterials under realistic conditions, liquids and ices, and novel electronic, photonic and battery materials. The instrument facilitates studies of the near-surface chemical composition, electronic and geometric structure of a variety of samples using X-ray photoelectron spectroscopy (XPS) and near-edge X-ray absorption fine-structure (NEXAFS) spectroscopy in the photon energy range from 170 eV to 2800 eV. The beamline provides a resolving power *h*ν/Δ(*h*ν) > 5000 at a photon flux > 10^10^ photons s^−1^ over most of its energy range. By operating the optical elements in a low-pressure oxygen atmosphere, carbon contamination can be almost completely eliminated, which makes the beamline particularly suitable for carbon *K*-edge NEXAFS. The endstation can be operated at pressures up to 100 mbar, whereby XPS can be routinely performed up to 30 mbar. A selection of typical data demonstrates the capability of the instrument to analyse details of the surface composition of solid samples under ambient-pressure conditions using XPS and NEXAFS. In addition, it offers a convenient way of analysing the gas phase through X-ray absorption spectroscopy. Short XPS spectra can be measured at a time scale of tens of seconds. The shortest data acquisition times for NEXAFS are around 0.5 s per data point.

## Abstract 2

The ambient-pressure endstation of the VerSoX beamline (B07) of Diamond Light Source provides a versatile facility for soft X-ray absorption and photoemission measurements in the mbar pressure range. A selection of typical data demonstrates the capability of the instrument to analyse details of the surface composition of solid samples and the environment gas under ambient-pressure conditions.

## 1. Introduction   

Over the last decade, there has been growing demand for a better understanding of the near-surface regions of a variety of samples in non-vacuum environments (Salmeron & Schlögl, 2008 ▸; Starr *et al.*, 2013 ▸), including heterogeneous catalysts under reaction conditions, pharmaceuticals and biomaterials in aqueous environments, electronic and photonic devices under realistic humidity conditions, and environmental studies on liquids and ices. All these areas of science have a common interest in the chemical nature and composition of the near-surface atomic layers. These are best characterized using soft X-ray photoelectron spectroscopy (XPS) and near-edge X-ray absorption fine-structure (NEXAFS) spectroscopy [also referred to as X-ray absorption spectroscopy (XAS)].

Efforts to design and build instruments enabling the study of such systems, both at synchrotrons and in the laboratory, have been described by a number of authors in recent publications (Ogletree *et al.*, 2002 ▸; Bluhm *et al.*, 2006 ▸, 2007 ▸; Ogletree *et al.*, 2009 ▸; Grass *et al.*, 2010 ▸; Bluhm, 2010 ▸; Schnadt *et al.*, 2012 ▸; Brown *et al.*, 2013 ▸; Crumlin *et al.*, 2013 ▸; Eriksson *et al.*, 2014 ▸; Kahk *et al.*, 2015 ▸; Edwards *et al.*, 2015 ▸; Knudsen *et al.*, 2016 ▸; Kerherve *et al.*, 2017 ▸; Arble *et al.*, 2018 ▸; Cai *et al.*, 2019 ▸; Novotny *et al.*, 2020 ▸). The ambient-pressure endstation of the new VerSoX (Versatile Soft X-ray) beamline B07 at Diamond Light Source is dedicated to XPS and NEXAFS experiments under near-ambient pressure conditions (up to 100 mbar). It enables studying the surface composition of heterogeneous catalysts under working conditions (as opposed to conventional *ex situ* characterization), characterization of biological and pharmaceutical samples under equilibrium water-vapour conditions at room temperature (32 mbar), and direct spectroscopy of surfaces of liquids. The beamline/endstation also enables studies of samples, such as those related to heritage conservation and polymeric materials, which do not require such high ambient pressures but are still often incompatible with ultra-high-vacuum (UHV) requirements due to outgassing.

In this paper we present the general considerations which guided the specifications and design of the beamline and endstation, their performance, and a selection of data demonstrating the capabilities of the instrument in terms of energy resolution and ambient-pressure environments.

## 2. General considerations   

The need for performing electron spectroscopy at pressures in the mbar range requires beamline specifications which deviate somewhat from typical soft X-ray beamlines. In particular the optimum photon energies for performing XPS experiments can be significantly higher than in UHV experiments.

Both soft X-rays and electrons interact very strongly with gas-phase molecules whereby their transmission α(*d*) through a layer of gas of thickness *d* is defined as the ratio of intensities, *I*(*d*) and *I*(0), after and before they have penetrated the layer. α(*d*) is determined by the Beer–Lambert law,
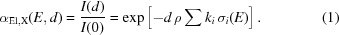
Depending on the type of radiation, *E* is either the photon energy, *h*ν, or the kinetic energy of the electrons, *E*_El_; σ_*i*_(*E*) is the energy-dependent atomic absorption cross section (for X-rays) or the inelastic scattering cross section (for electrons). The index *i* runs over all constituent atoms of the gas molecules and *k*_*i*_ is their relative abundance in the gas mixture; ρ = *N*/*V* is the density of molecules of the gas, which depends on the pressure *p* through the ideal gas equation,
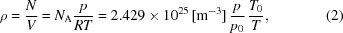
with *N*_A_ being Avogadro’s number, *T*_0_ = 298.15 K, and *p*_0_ = 1 bar. It is obvious from equation (1) that the increased attenuation through high pressures can be compensated by reducing the path length *d*.

**Figure 1:**
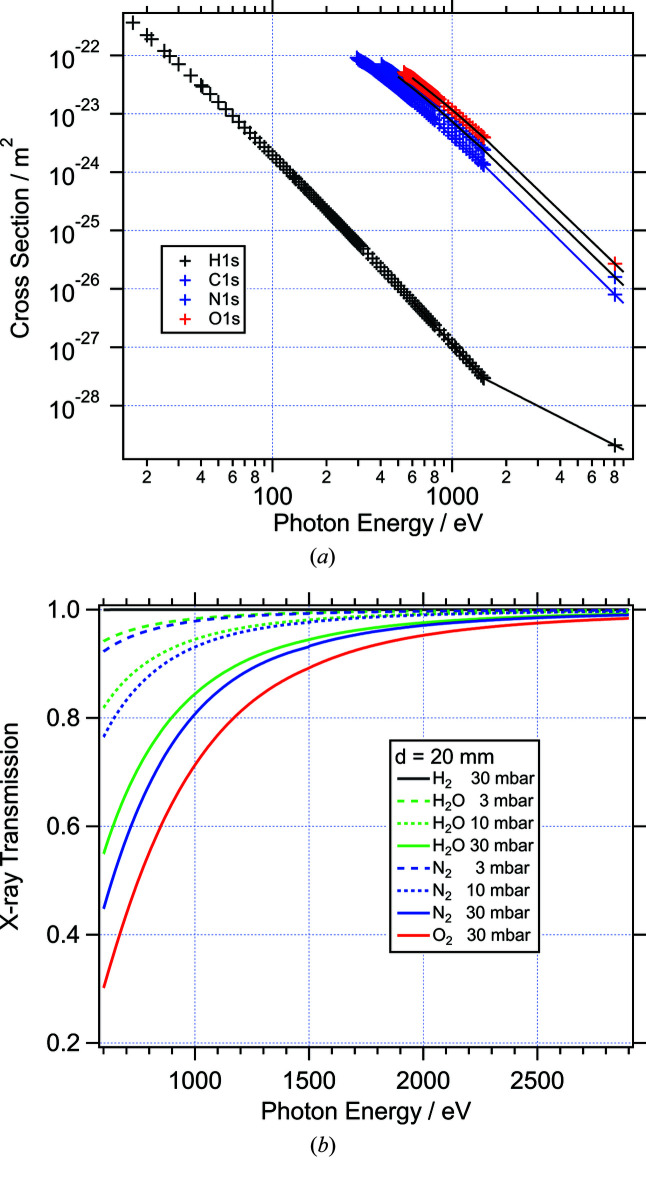
(*a*) 1*s* subshell cross sections for H, C, N, O atoms extracted from Yeh & Lindau (1985 ▸). The solid lines indicate parametrizations of the data points for *E*_ph_ > 530 eV. (*b*) Transmission of X-rays through a length of 20 mm at different pressures for H_2_O, H_2_, N_2_, and O_2_.

Absorption cross sections for light atoms have a strong energy dependence and range from around 10^−22^ m^2^ to 10^−28^ m^2^ in the soft X-ray range. Fig. 1 ▸(*a*) shows atomic absorption cross sections for common gas constituents as a function of photon energy (Yeh & Lindau, 1985 ▸). For the low-*Z* atoms included in the figure, the total photo-absorption cross section is dominated by the absorption of the 1*s* subshell above the 1*s* absorption edge. Therefore it is sufficient to consider only these for photon energies above 530 eV (O 1*s*). Fig. 1 ▸(*b*) shows the transmission for typical gases (H_2_, H_2_O, N_2_, O_2_) calculated according to equation (1). The path length, *d*_X_, used to calculate the transmission curves is 20 mm, equal to the distance between the beamline entrance and the sample in the VerSoX ambient-pressure endstation (see below). The transmission curves were produced using a parametrization of the cross-section data, as indicated by the solid lines in Fig. 1 ▸(*a*). From Fig. 1 ▸(*b*) it is clear that at pressures above 3 mbar the gas-phase absorption of soft X-rays with energies below 1000 eV is already very significant for most gases, except H_2_.

**Figure 2:**
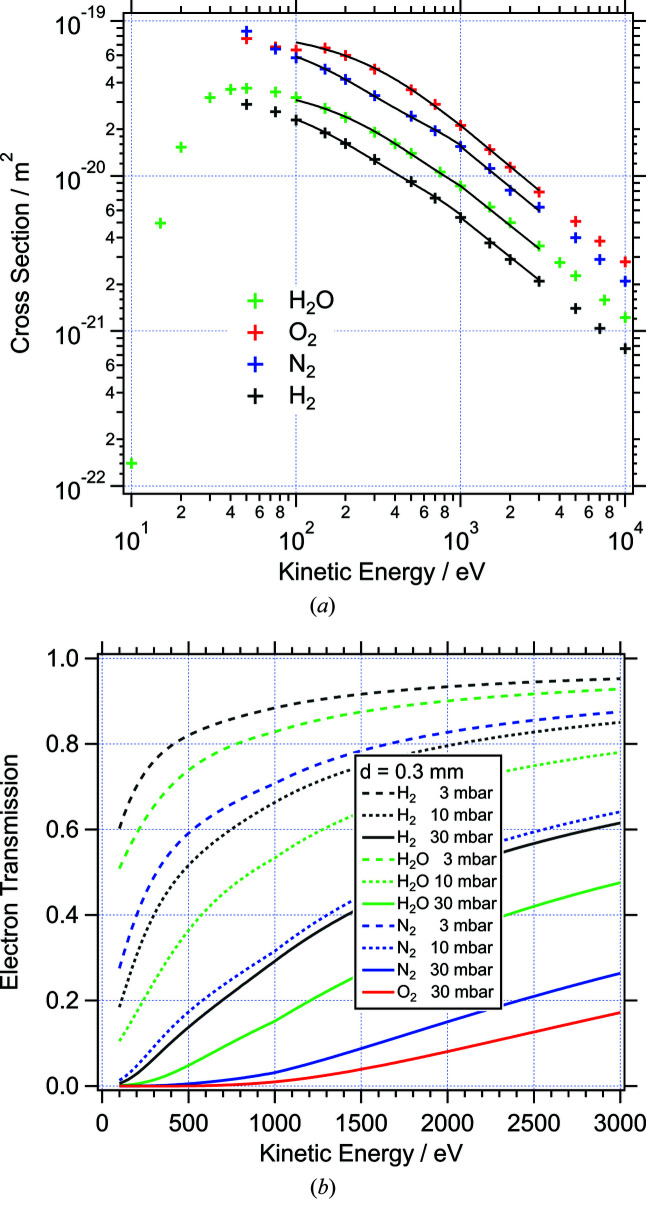
(*a*) Inelastic electron scattering cross-section of common gas molecules (H_2_O, H_2_, N_2_, O_2_) as a function of electron kinetic energy. Data extracted from Muñoz *et al.* (2007 ▸) (H_2_O), Lawton & Phelps (1978 ▸) (O_2_), Phelps & Pitchford (1985 ▸) N_2_, and Buckman & Phelps (1985 ▸) (H_2_). The solid lines indicate parametrizations of the data points for *E*_kin_ > 100 eV. (*b*) Transmission of electrons over a distance of 0.30 mm at different pressures of the above gases.

**Figure 3:**
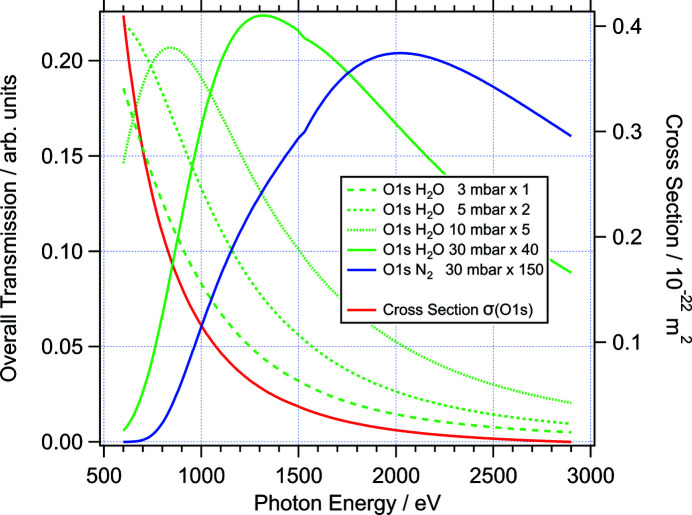
Overall photoelectron transmission under ambient pressure conditions. See text for details.

The attenuation of electrons shows a similar qualitative behaviour as a function of kinetic energy, although the numerical values for the inelastic scattering cross sections are typically about two orders of magnitude larger than for X-ray absorption [see Fig. 2 ▸(*a*)] for a number of common gas molecules (Lawton & Phelps, 1978 ▸; Phelps & Pitchford, 1985 ▸; Buckman & Phelps, 1985 ▸; Muñoz *et al.*, 2007 ▸). Thus electron attenuation is the limiting factor in ambient-pressure experiments and the path length for electrons penetrating the gas phase must be kept in the sub-millimetre range. This can be achieved by small analyser apertures and differentially pumped lens systems (Salmeron & Schlögl, 2008 ▸; Salmeron *et al.*, 2009 ▸; Bluhm, 2010 ▸; Starr *et al.*, 2013 ▸; Kahk *et al.*, 2015 ▸). Fig. 2 ▸(*b*) shows the transmission of electrons through *d*_El_ = 0.3 mm of gases of the same type as above. Similarly to photons, the attenuation depends critically on the type of ambient gas. Especially at low kinetic energies, below 500 eV, the heavier gases N_2_ and O_2_ are almost unpenetrable with transmissions below 1% at 30 mbar. As a consequence, higher kinetic energies must be chosen for these pressures. In photoelectron spectroscopy, this can be achieved by using higher photon energies, as the kinetic energy of photoelectrons for a given subshell increases linearly with the photon energy. On the other hand, the cross section for the excitation of a photoelectron, σ_sample_(*h*ν), decreases as a function of the photon energy above the absorption threshold. Ultimately, this outweighs the intensity gain through lower attenuation at high photon/kinetic energies. In order to optimize the photoelectron signal from samples in a gas atmosphere, one therefore has to consider the product of transmission and excitation cross section for a given photon energy,
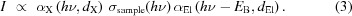
The maximum of this product defines the optimum photon energy at which an ambient-pressure experiment should ideally be carried out. Obviously other factors also play important roles, such as resolution and transmission of analyser and/or beamline. Fig. 3 ▸ demonstrates the photon energy dependence of O 1*s* photoelectrons (binding energy 530 eV) at different pressures of water vapour and nitrogen, according to equation (3). The red curve in Fig. 3 ▸ shows the O 1*s* photoelectron excitation cross section as a function of photon energy (right axis). The four green curves and the blue curve are examples of the product in equation (3) for 1, 5, 10, and 30 mbar of water vapour and 30 mbar of nitrogen gas, using the same path length for X-rays (*d*_X_ = 20 mm) and electrons (*d*_El_ = 0.3 mm) as before.

At 3 mbar H_2_O the overall transmission function still has essentially the same energy dependence as the excitation cross section. At 5 mbar significant deviations between the two curves are observed; however, the maximum is still near the excitation threshold of 530 eV. For 10 and 30 mbar the maxima shift towards higher photon energies, around 850 and 1300 eV, respectively, and the overall intensities drop significantly (note that the corresponding curves in Fig. 3 ▸ are multiplied by 5 and 40, respectively). For the heavier gas N_2_ the optimum photon energy at 30 mbar shifts to around 2000 eV. In this case, the signal is essentially zero for photon energies below 700 eV, which would normally be used for O 1*s* photoelectrons in UHV environments.

As a general conclusion, higher pressures above 5 mbar require photon energies at the upper end of the soft X-ray range. These considerations were taken into account at the conceptional design stage of the beamline; therefore the energy range was extended as far as possible at the upper end.

## 3. Beamline   

**Figure 4:**
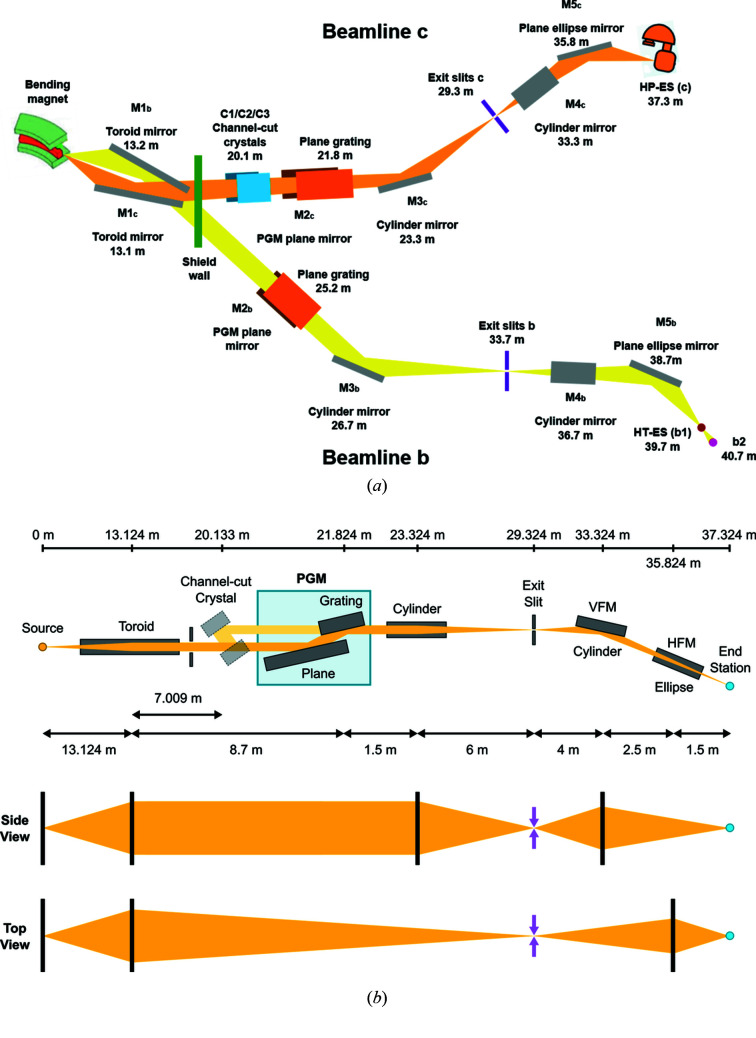
(*a*) General layout of the beamline including both branchlines B and C. (*b*) Layout of the optical elements of branch C.

Beamline B07 consists of two branchlines, B and C, each of which has its own source and monochromator. Therefore they can be operated simultaneously and independently [see Fig. 4 ▸(*a*)]. Here we concentrate on branch C, which provides radiation for the ambient-pressure endstation.

### 3.1. Beamline design   

The overall design criterion for branch C was to obtain maximum photon flux and reasonable energy resolution (*E*/Δ*E* > 5000) over a wide range of photon energies, from 250 eV to 2800 eV. Fig. 4 ▸(*a*) shows an overview of the beamline design. The source is a 1.4 T bending magnet emitting horizontally polarized radiation with a fan of approximately 30 mrad horizontal width. Two segments of the fan, at 6–8 mrad and 22–24 mrad, are reflected into the two branchlines B and C by a pair of mirrors, M1b and M1c, respectively, at distances 13.2 m and 13.1 m from the bending magnet source. The vertical acceptance angle of each mirror is 0.4 mrad, which limits the photon flux into the monochromator to values between 1 × 10^13^ and 3 × 10^13^ photons s^−1^ (0.1% bandwidth)^−1^ over the energy range 250–2800 eV at 300 mA ring current. The acceptance angles were defined by the maximum mirror size available from the manufacturers (optics surface 1370 mm × 15 mm). The M1 mirrors are toroids which collimate the beam in the vertical direction and focus horizontally onto the exit slit of their monochromators. Accurate control of the mirror temperature allows compensation of mirror distortions and fine adjustment of the focal length (Hand *et al.*, 2019 ▸). The monochromators are of the collimated plane grating monochromator type (Follath & Senf, 1997 ▸) (cPGM, manufactured by FMB Berlin) with a plane mirror, M2b/c, directing the collimated beam onto the grating and a sagitally focusing cylindrical mirror, M3b/c, focusing the monochromatic beam vertically onto the exit slit located 8.5 m and 7.5 m, respectively, downstream. The cPGM of branch C has three gratings with 400, 600, and 1200 lines mm^−1^, designed to provide an energy resolving power *h*ν/Δ(*h*ν) > 5000 over the energy range 250–2500 eV. The use of incident collimated light allows working with variable *c*_ff_ and thus optimizing the suppression of second and higher diffraction orders. As an alternative to the cPGM, branch C also has three channel-cut Si crystal monochromators (Berman *et al.*, 1985 ▸) which can be inserted into the beamline before the cPGM, bypassing M2c while the gratings are retracted from the path of the synchrotron radiation [see Fig. 4 ▸(*b*)]. These will eventually provide monochromatic radiation of 2000, 2250 and 2500 eV with higher resolution and flux than the cPGM at these energies. A pair of refocusing mirrors, M4c (vertical, VFM) and M5c (horizontal, HFM), located after the exit slit, focus the monochromatic beam onto the sample position. The theoretical minimum beam size is 50 µm × *y*_gap_, where *y*_gap_ is the vertical size of the exit slit. The most important parameters of the key optical elements of branch C are listed in Table 1 ▸. All motions of optical elements, shutters, diagnostics and vacuum control are fully integrated into the EPICS (Experimental Physics and Industrial Control System, https://epics-controls.org/) and GDA (Generic Data Acquisition, http://www.opengda.org/) control environment of the beamline.

A key improvement in the vacuum design of the beamline is that all optical elements are operated in an atmosphere of 1 × 10^−8^ to 5 × 10^−8^ mbar oxygen (controlled via leak valves) which is approximately two orders of magnitude higher than the base pressure of the vacuum vessels. Oxygen is activated by the incident X-rays near the mirror surfaces and reacts with carbon to form volatile compounds, CO or CO_2_, thus avoiding the build-up of carbon deposits (Risterucci *et al.*, 2012 ▸).

### 3.2. Beamline performance   

**Figure 5:**
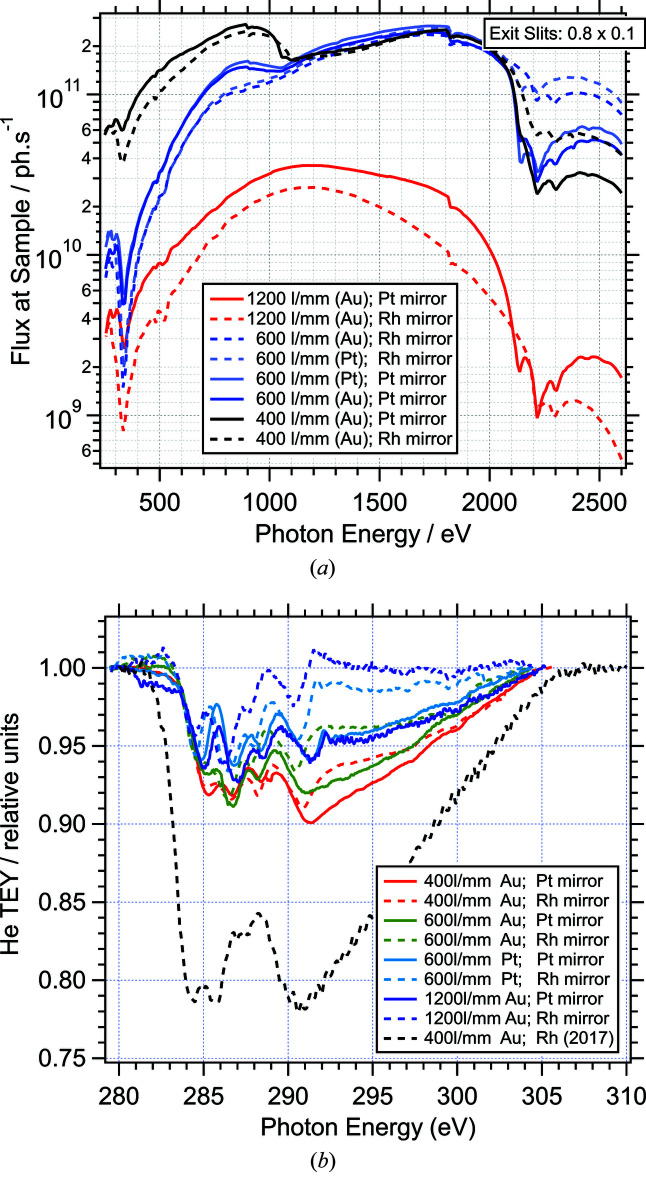
(*a*) Transmission of beamline B07-C for all combinations of cPGM mirrors and gratings; the photon flux was measured using a photodiode at the sample position. (*b*) Beamline transmission in the energy range of the carbon *K*-edge; the photon flux was measured through total electron yield (TEY) from He gas in the endstation.

Up to now, branchline C has been used for experiments in the energy range from 170 eV (boron *K*-edge) to 2700 eV (molybdenum *L*_2_-edge). Fig. 5 ▸(*a*) shows the transmission of the beamline between 250 eV and 2600 eV for all combinations of cPGM mirrors and gratings. The photon flux (with exit slit gap 0.1 mm) was measured using a photodiode after M5c. Between 500 eV and 2100 eV a flux of around 1 × 10^11^ photons s^−1^ can be achieved with the 400 and 600 lines mm^−1^ gratings; the high-resolution 1200 lines mm^−1^ grating delivers about an order of magnitude less flux. At higher energies the flux is significantly reduced with all gratings and has several dips due to absorption by the Au and Pt coating material of the optical elements. Significant other absorption features in the transmission spectrum are around 332 eV (Rh *M*_4, 5_) and 1822 eV (Si *K*). Importantly, features due to carbon *K*-edge absorption around 285 eV, which are very prominent in most soft X-ray beamlines, are reduced to around 10%. This is the effect of operating the optical elements in an oxygen atmosphere. The transmission curves for different mirror/grating combinations in this energy region are shown in Fig. 5 ▸(*b*). They were measured in March 2019 using the total electron yield (TEY) signal of He gas in order to avoid features from carbon contamination on the photodiode. The comparison with data from August 2017 (at the start of user operation), which are also included in the figure, show that the ‘carbon dip’ is actually reduced over time. The small dip due to oxygen *K*-edge absorption around 535 eV (∼10%) is not significantly bigger than in comparable beamlines.

N_2_ absorption spectra recorded in the endstation confirm a maximum resolving power, *h*ν/Δ(*h*ν), of at least ∼7700 (400 lines mm^−1^, exit slit opening 0.0125 mm; see Fig. S1 of the supporting information). The beam size at the sample position was measured using the edge of a thin mica sheet (see Fig. S2 of the supporting information). The vertical size depends on the opening of the exit slit with values between 0.06 mm (exit slit 0.025 mm) and 0.10 mm (exit slit 0.200 mm); the horizontal size is 0.09 mm.

## 4. Endstation   

**Figure 6:**
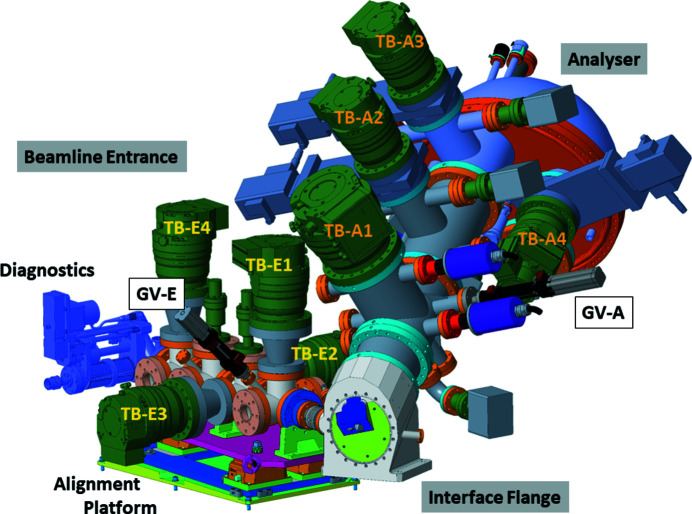
Overview of the arrangement of analyser and beamline entrance. See text for details.

**Figure 7:**
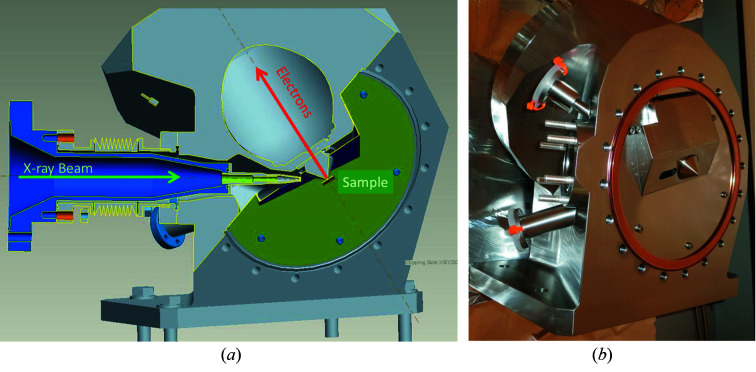
Details of the interface flange. (*a*) Detailed drawing and (*b*) photograph.

Fig. 6 ▸ shows an overview drawing of the VerSoX endstation without the actual ambient-pressure sample vessel. It consists of a differentially pumped beamline entrance and a differentially pumped hemispherical analyser, which both meet in a single interface flange. A more detailed drawing and a photograph of the interface flange are shown in Figs. 7 ▸(*a*) and 7(*b*). The sample chamber is attached to the front of the interface flange, thus allowing exchange of different sample environments while preserving the alignment between beamline and analyser.

### 4.1. Beamline entrance   

The windowless beamline entrance (manufactured by SPECS, Berlin, Germany) has four differential pumping stages, each pumped by a turbomolecular pump (TB-E1-4 in Fig. 6 ▸). The last aperture (nearest to the sample) has a diameter of 0.3 mm. It is mounted on a nozzle protruding into the interface flange, at around 20 mm from the sample position. The apertures separating the pumping stages have bigger apertures of 2.5 to 5.0 mm, corresponding to the diameter of the beam at the respective positions. A gate valve (GV-E in Fig. 6 ▸) allows separating the first pumping stage from the high-vacuum stages. Thus the endstation can be vented while protecting the vacuum in the upstream stages. The apertures are electrically isolated, such that their drain current can be measured for alignment and calibration purposes. Tests with He gas confirmed that a pressure in the 10^−9^ mbar range can be maintained in the M5c mirror vessel while the pressure at the sample is up to 100 mbar.

The diameter of the last aperture, 0.3 mm, is big enough for the beam to pass through without inducing significant photocurrent. Therefore it can be used as electron collector for X-ray absorption spectroscopy of the gas in the analyser chamber if a suitable positive bias voltage is applied (typically +36 eV). This feature enables very quick and straightforward characterization of the beamline and can be used for *I*_0_ measurements using the sample chamber as ionization chamber or for characterizing the gas composition in the chamber.

An array of diagnostics tools is available before the first aperture, near mirror M5c. This includes a gold mesh (61% transparency) and a photodiode (Hamamatsu G1127) for *I*_0_ measurements, and a screen for beam shape diagnostics.

### 4.2. Analyser   

The electron energy analyser is a ‘PHOIBOS 150 NAP’ hemispherical analyser supplied by SPECS, Berlin, Germany (Bluhm *et al.*, 2007 ▸). It is fitted with a pre-lens, which is also the first of four differential pumping stages (TB-A1-4 in Fig. 6 ▸), and a 2D delay-line detector by Surface Concept, Mainz, Germany. Similar to the beamline entrance, a gate valve, GV-A, can separate the pre-lens from the other pumping stages, thus protecting the vacuum in the UHV section of the analyser when the endstation is vented. The entry cone of the pre-lens is integrated into the interface flange (see Fig. 7 ▸). It is interchangeable and isolated from ground. It can be biased (typically +36 V) to pull electrons into the analyser, which leads to about 10% increase in the signal without affecting the measured kinetic energy, and it can be used (simultaneously if required) as electron collector for X-ray absorption measurements or for other purposes. Currently, the instrument is operated with a cone aperture of 0.3 mm diameter, which is slightly larger than the footprint of the beam at normal emission (∼0.2 mm) and allows pressures up to 100 mbar in the analysis chamber while keeping the pressure in the detector section of the analyser below 10^−6^ mbar. The first two differential pumping stages contain a quadrupole mass spectrometer each for the analysis of the sample environment.

The analyser axis is at an angle of 60.1° with respect to the beam and tilted 30° with respect to the horizontal, *i.e.* close to the magic angle with respect to the polarization vector. The entire assembly of interface flange and analyser is mounted on a ‘sledge’ which can be moved parallel to the analyser axis by a stepper motor. This way the cone-to-beam/sample distance can be accurately adjusted (reproducibilty  ±10 µm) without having to re-align the endstation, once the beam and the analyser axis have been adjusted such that they intersect in one point. The typical working distance between cone and sample is 0.2–0.3 mm.

The interface flange and the sample environments are made from stainless steel. Compensation of residual magnetic fields is achieved via three pairs of Helmholtz coils, which are individually controlled as a function of electron energy through the control unit of the electron energy analyser. In normal operation, no entrance slit is used in the hemispheres; instead the cone aperture is limiting the size of the electron beam entering the analyser. Spectra can be recorded either in scanned or snapshot mode. In the latter the retarding voltage is kept constant and the intensities along the energy-dispersive direction of the 2D detector are directly assigned to the respective kinetic energies. The maximum width of a spectrum measured in this mode is about 12% of the pass energy (typically 0.6 to 6 eV). In the scanned mode the retarding voltage is varied and the intensity of each point in the spectrum is determined by integrating the signals of the respective positions recorded on the 2D detector in each step.

### 4.3. Sample environments   

**Figure 8:**
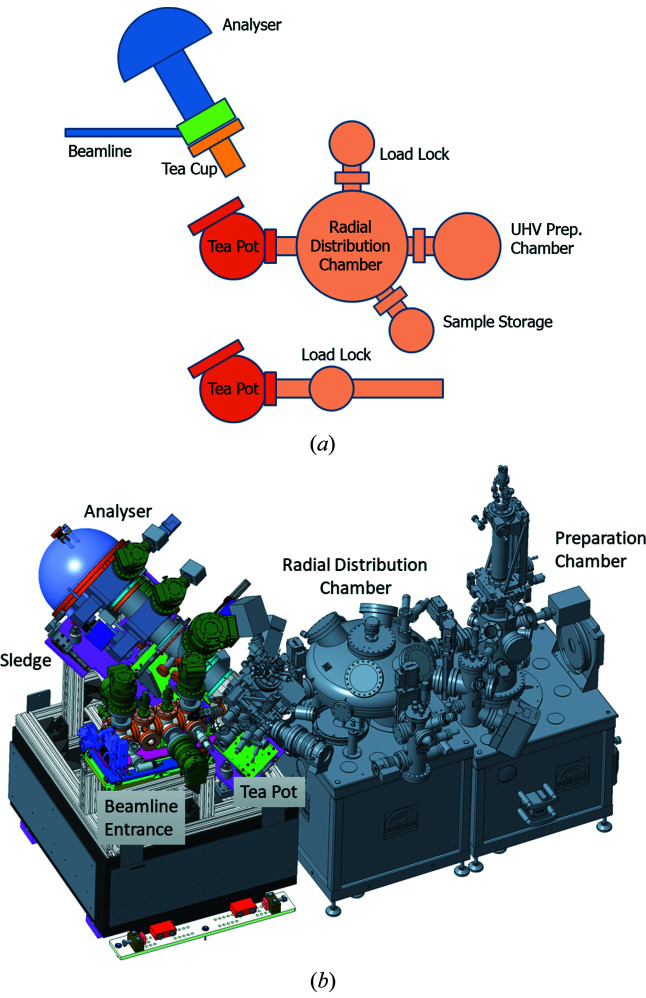
(*a*) Schematics of the sample chambers available at VerSoX. (*b*) 3D drawing of the endstation with Tea Pot, radial distribution chamber, and UHV preparation chamber.

As mentioned before, the design of the interface flange allows exchanging sample chambers without having to re-adjust the alignment between beamline and analyser. Currently the endstation offers two different sample environments, a small UHV-compatible chamber with sample transfer system (‘Tea Pot’) and a smaller reaction cell with fast entry system (‘Tea Cup’). The different configurations are schematically depicted in Fig. 8 ▸.

#### 4.3.1. Tea Pot   

The Tea Pot sample chamber is fitted with a five-axis manipulator and sample receiver from Prevac, Rogow, Poland. Base pressures of 10^−9^ mbar are typically reached after a short bakeout (using two internal halogen lamps). The sample receiver is compatible with a variety of Prevac ‘PTS’-style sample holders for different temperature and pressure ranges (see the supporting information for more details). It can be cooled by air or liquid nitrogen down to a temperature of 150 K. Resistive heaters and K-type thermocouples are integrated in the sample holders, which allow maximum temperatures up to 1000 K, depending on the type of sample holder and the chamber pressure. The heaters are designed such that magnetic fields at the sample position are minimal. Typical intensity variations between heating on and off are around 10%. The sample receiver also has spare electrical contacts allowing, for example, biasing the sample. Sample holders can be easily adapted to hold liquid cells or other special sample environments.

The simplest configuration of the Tea Pot is shown at the bottom of Fig. 8 ▸(*a*). It consists of the analyser chamber and an entry lock, which is directly attached. Samples can be transferred from air, a glove bag, or a vacuum suitcase within less than 5 min, either in vacuum (<10^−6^ mbar) or in a controlled gas atmosphere matching that of the experiment. This setup is best suited for samples which can be prepared *ex situ*; the only treatment *in situ* is through heating and ambient gases.

The middle of Fig. 8 ▸(*a*) and the 3D drawing in Fig. 8 ▸(*b*) depict an extended configuration where the Tea Pot chamber is connected to a radial distribution chamber. This enables *in vacuo* transfer of samples between the vacuum load lock, a UHV sample storage chamber, holding up to six samples, a UHV sample preparation chamber, and the analyser chamber. Each can be isolated via gate valves and operated/vented independently. The UHV preparation chamber is equipped with a sputter gun, a LEED system, up to three evaporator sources (mounted behind gate valves for easy exchange and maintenance), and two leak valves for gas dosing. This setup is suited for samples which need to be prepared in UHV before they are studied in ambient-pressure environments. It allows treating one sample in the UHV preparation chamber while experiments are performed with another sample in the analyser chamber. Sample transfer is only possible in vacuum.

#### 4.3.2. Tea Cup   

**Figure 9:**
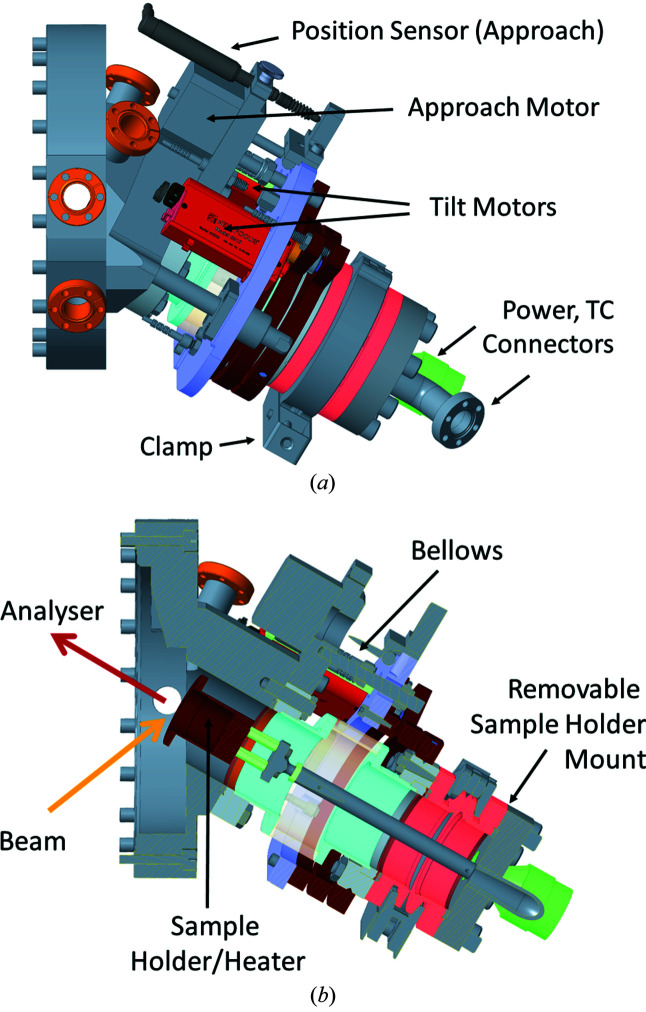
(*a*) Side view of the Tea Cup chamber. (*b*) Cut through the Tea Cup chamber, showing the position of the sample.

The Tea Cup reaction chamber was developed in-house. This system is mainly dedicated to experiments with powder catalyst samples in reactive and contaminating gas environments at pressures >0.1 mbar. The overarching design principle was to keep the vessel volume small (0.7 L), in order to enable fast switching of environment gases, and to keep the costs low, such that different Tea Pots can be provided for experiments with different gases, to avoid cross-contamination. A schematic drawing is included at the top of Fig. 8 ▸(*a*); Fig. 9 ▸ shows more detailed drawings. The sample holder (see supporting information) includes a button heater and temperature sensor (by Heatwave Labs, USA; maximum temperature 1000°C). It is mounted on a small manipulator which allows linear travel along the analyser axis and tilting in the two directions perpendicular to the analyser axis, which enables lateral displacements of ±1 mm with negligible change in distance from the analyser. The sample holder can easily be replaced by an electrochemical cell or other more complex sample stages.

#### 4.3.3. Computer control   

Temperature control of the two available sample holders, all manipulator/analyser motions, and all endstation signals (sample current, analyser, diagnostics, *etc*.) are fully integrated in the EPICS and GDA controls and data acquisition environment of the beamline. This enables very versatile scripting of experimental procedures [EPICS (https://epics-controls.org/) and GDA (http://www.opengda.org/)]. The accuracy of sample and analyser position is typically less than 10 µm. A gas dosing system is under construction, which will also be integrated in the EPICS/GDA environment. Currently the gas pressure and composition is controlled manually via leak valves.

## 5. Experiments   

In the following section we report selected experiments which demonstrate the capabilities of the beamline and endstation and characterize some of the key parameters.

### 5.1. Energy resolution of the beamline: gas-phase absorption spectra   

**Figure 10:**
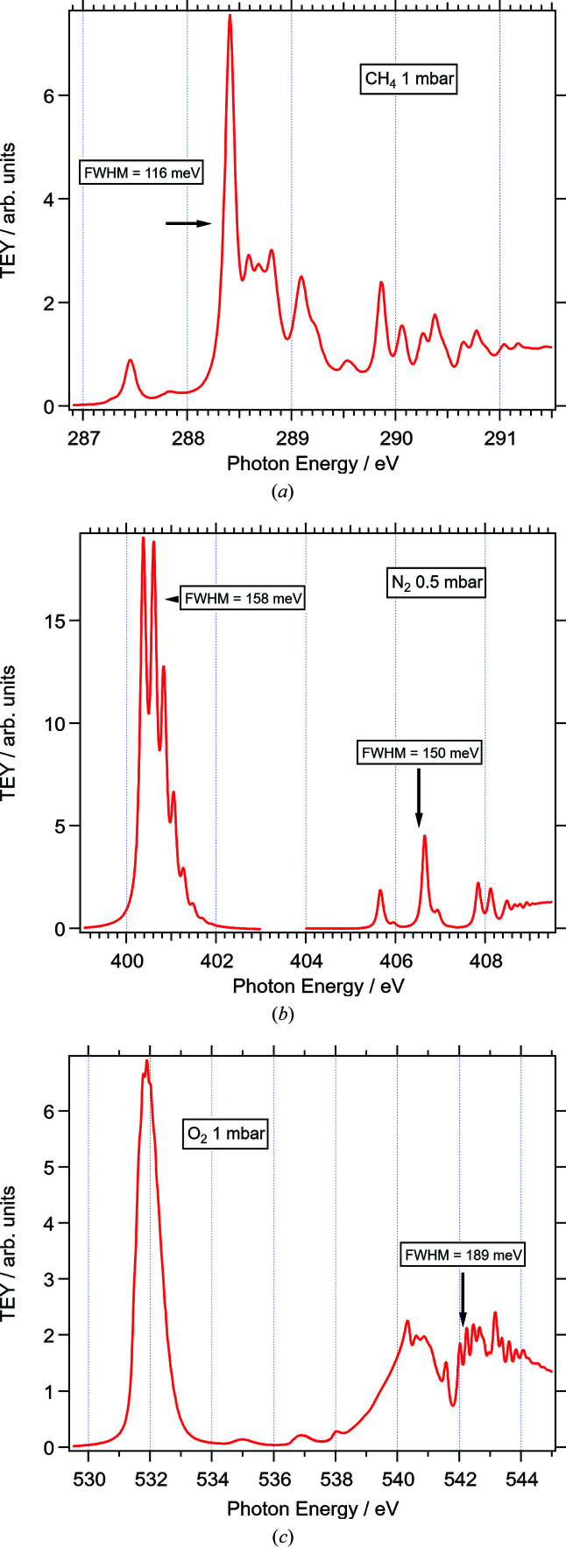
Gas-phase NEXAFS spectra of (*a*) methane, (*b*) N_2_, (*c*) O_2_ measured through the TEY signal on the beamline entry nozzle. The half width of some of the peaks are indicated in the spectra.

Figs. 10 ▸(*a*)–10(*c*) show high-resolution gas-phase X-ray absorption spectra of the C, N, and O *K*-edges of methane, N_2_, and O_2_, respectively, measured after backfilling the Tea Pot chamber to pressures between 0.5 and 1 mbar. In order to achieve the best combination of flux and resolution, the beamline was operated with the 600 lines mm^−1^ grating, the smallest exit slit, 0.012 mm, and the photon energy was scanned in steps of 0.005 or 0.010 eV. The nozzle of the beamline entrance was biased +36 V and connected to a electrometer (Stanford Research Systems SR570) to measure the secondary TEY while repelling positive ions. Typical TEY currents were in the nA range for the above pressures. Data acquisition times were typically 0.5 s per data point, which is close to the limit imposed by the monochromator motion and settling time. The spectra agree in every detail with those published previously (Ueda *et al.*, 1995 ▸; Urquhart & Gillies, 2005 ▸; Prince *et al.*, 1998 ▸; Kato *et al.*, 2007 ▸; Feifel *et al.*, 2008 ▸), when the energy axis is aligned accordingly (note, the photon energy axes of the spectra are not calibrated to match literature values of the respective absorption lines). Some of the measured line widths (full width of half-maximum, FWHM) of individual peaks are indicated in Fig. 10 ▸. These are at the same levels as in spectra published previously from high-resolution beamlines and close to the lowest published estimates for the natural line widths of 94 meV for methane (Tronc *et al.*, 1979 ▸), 115 meV for N_2_, (Prince *et al.*, 1999 ▸) and 149 meV for O_2_ (Prince *et al.*, 1999 ▸). Fitting individual peaks with Voigt functions using these latter values as Lorentzian line widths renders Gaussian broadening of 50, 76, and 85 meV for the C, N and O edges. This corresponds to resolving powers *h*ν/Δ(*h*ν) between 5260 and 6260 in this energy range.

### 5.2. Combined energy resolution of analyser and beamline: X-ray photoelectron spectroscopy   

**Figure 11:**
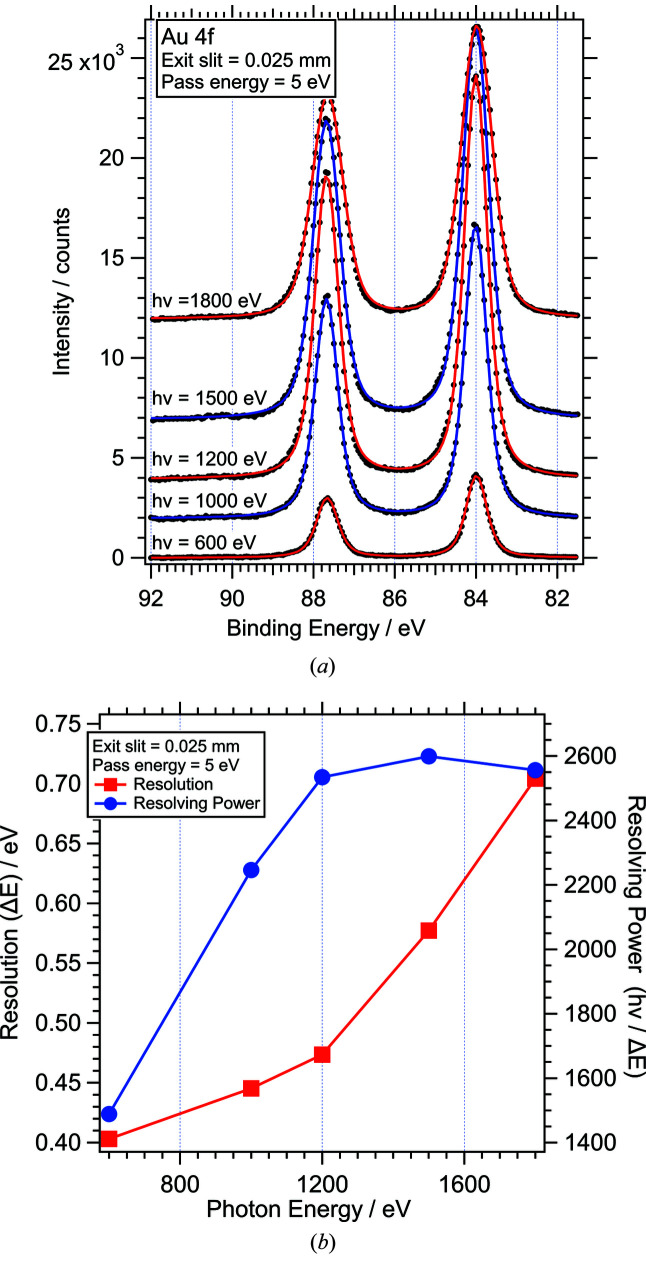
(*a*) Au 4*f* spectra recorded at different photon energies (exit slit = 0.025 mm; pass energy = 5 eV); data points (dots) and fits (lines) as discussed in the text. (*b*) Combined energy resolution Δ*E* (squares) and resolving power *h*ν/Δ*E* (circles) of beamline and analyser

Fig. 11 ▸(*a*) shows a series of Au 4*f* photoelectron spectra recorded from a sputtered polycrystalline gold foil with different photon energies, using an exit slit opening of 0.025 mm (600 lines mm^−1^) and analyser pass energy of 5 eV. The pass energy and exit slit settings are not the lowest possible but represent typical values used to achieve high resolution with reasonable intensity. Energy calibration was applied by shifting the binding energy axis such that the Au 4*f*_7/2_ peak is at 84.0 eV. No other data treatment or normalization was applied, thus variations in the signal intensity are determined by variations in the excitation cross section and the transmission of beamline and analyser. The combined energy resolution of beamline and analyser was determined by fitting the Au 4*f* spectra with Voigt functions of fixed Lorentzian and variable Gaussian width. The former full width at half-maximum (FWHM_L_) was determined from fits to the best resolved spectra (*h*ν = 600 eV, FWHM_L_ = 0.340 eV/0.365 eV for Au 4*f*_7/2_/4*f*_5/2_); the latter was constrained to be the same for both peaks and was used as a measure for the experimental energy resolution Δ*E*. Fig. 11 ▸(*b*) shows the combined energy resolution Δ*E* and resolving power *h*ν/Δ*E* of beamline and analyser as a function of photon energy. At low photon energies, up to about 1000 eV, the overall resolution is predominantly determined by the analyser and inhomogeneity of the sample. For *h*ν = 600 eV, increasing the exit slit from 0.025 to 0.100 mm leads to only a small increase of Δ*E*, from 0.45 to 0.51 eV. Above 1000 eV, the resolution shows a strong dependence on the exit slit opening, indicating that the overall resolution is now determined by the beamline. At *h*ν = 1500 eV, the same change of the exit slit leads to an increase of Δ*E* from 0.62 to 1.28 eV. It is important to note that instrument resolution is not the only contribution to the Gaussian width. Other contributions are due to the inhomogeneity of the sample, *e.g.* surface core level shifts from different surface orientations or small levels of contamination. Therefore the values of Δ*E* in Fig. 11 ▸(*b*) have to be seen as upper limits, especially for the low photon energies, where Δ*E* is significantly higher than the expected resolution of beamline [∼0.12 eV for *h*ν = 600 eV, assuming *h*ν/Δ(*h*ν) = 5000] and analyser (∼0.005 eV for pass energy 5 eV, assuming a resolving power of 1000, as stated by the manufacturer). Gas-phase spectra show significantly lower Gaussian broadening close to the expected values, *e.g.* Δ*E* = 0.21 eV, of which 0.03 eV is due to thermal broadening (Baltzer *et al.*, 1993 ▸), for the O 1*s* lines of O_2_ recorded with *h*ν = 750 eV and optimized beamline and analyser settings (see Fig. S3 of the supporting information).

### 5.3. X-ray photoelectron spectroscopy at ambient pressures   

**Figure 12:**
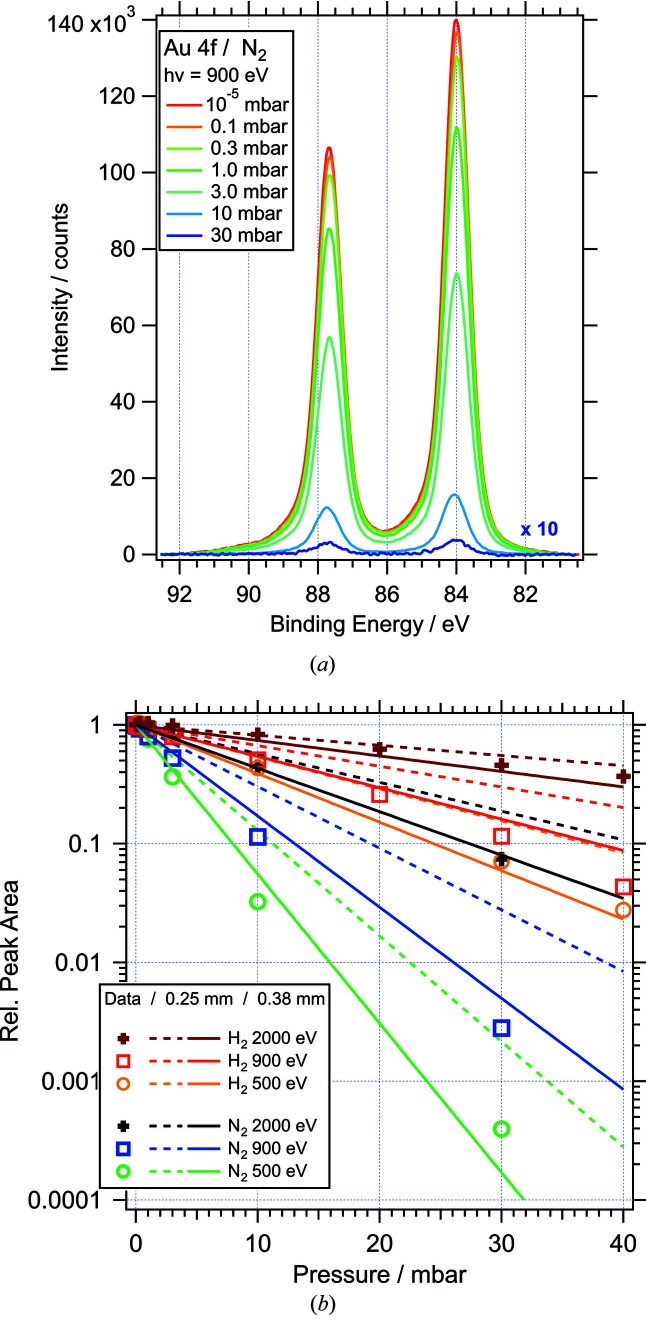
(*a*) Au 4*f* spectra recorded at different N_2_ pressures between 10^−5^ and 30 mbar (photon energy = 900 eV; exit slit = 0.05 mm; pass energy = 20 eV). (*b*) Au 4*f* peak intensity as a function of N_2_ and H_2_ pressure for different photon energies. Symbols: integrated peak area of the measured Au 4*f* signal; dashed/solid lines: model calculation for photoelectron path length of 0.25/0.38 mm.

The attenuation of the photoelectron signal in gas was characterized by measuring Au 4*f* spectra for different pressures of N_2_ and H_2_. Fig. 12 ▸(*a*) shows a selection of spectra for different N_2_ pressures collected with a photon energy of 900 eV (kinetic energy ≃ 814 eV). The data acquisition time for each spectrum was around 8 min. For the highest pressure, 30 mbar, the height of the Au 4*f*_7/2_ peak is ∼400 counts (0.3% of the UHV signal), which is sufficient for most data analysis. Signals with lower photoemission cross sections can still be routinely detected at this pressure if the data acquisition time is adjusted accordingly. The complete set of data is summarized in Fig. 12 ▸(*b*). The symbols represent the Au 4*f* peak area for photon energies of 500, 900 and 2000 eV (kinetic energies ≃ 414, 814, 1914 eV) and pressures between 0.1 and 40 mbar. The areas are normalized with respect to the signal measured under vacuum conditions (<10^−5^ mbar), thus eliminating any intensity variations due to different excitation cross sections or beamline/analyser transmission at different photon energies. In the semi-logarithmic plot of Fig. 12 ▸(*b*) the data points for a given photon energy and gas type line up on straight lines. The only exception is the data set for N_2_ gas and *h*ν = 500 eV, where the high-pressure signal is difficult to distinguish from the noise. The data therefore show the Lambert–Beer-type behaviour expected from equation (1). The lines in the figure represent the normalized transmission, according to equation (3), expected for Au 4*f* photoelectrons penetrating the gas phase over distances of 0.25 mm (dashed lines) and 0.38 mm (solid lines), in both cases using cross-section data from Phelps & Pitchford (1985 ▸) and Buckman & Phelps (1985 ▸). The distance between analyser cone and sample was 0.25 mm for these measurements, which was confirmed by moving the sample towards the cone until electrical contact was established. This distance, however, does not take into account the depth of the cone aperture, which is specified as 0.08 mm by the manufacturer and needs to be added to obtain the path length to the low-pressure side of the cone aperture, *i.e.* 0.33 mm. The best overall agreement with the data is achieved by modelling the transmission with an effective photoelectron path length of 0.38 mm [solid lines in Fig. 12 ▸(*b*)]. This effective path length accounts for the attenuation of the X-rays in the gas phase, as well as the tail of decaying gas density beyond the aperture. Several recent studies have proposed models for the pressure distribution around the analyser aperture (Ogletree *et al.*, 2002 ▸; Bluhm, 2010 ▸; Kahk *et al.*, 2015 ▸). Despite differences in the details, all models agree that the gas pressure does not change by more than 20% up to a distance of half the diameter of the aperture and rapidly decreases to below 20% within the same distance inside the first differential pumping stage. Our findings are well within these models.

### 5.4. Temperature-programmed X-ray photoelectron spectroscopy   

**Figure 13:**
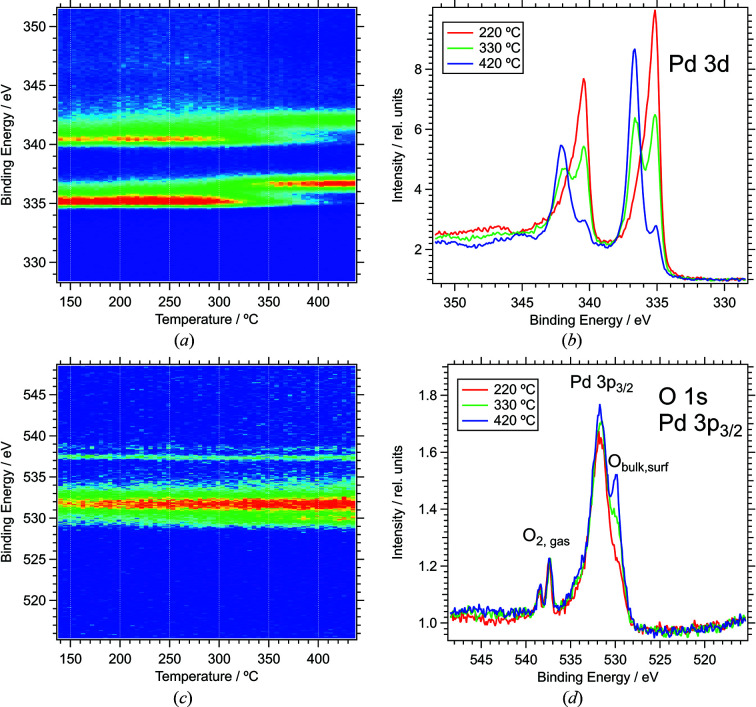
Temperature-programmed XPS spectra during heating of a Pd foil in 1 mbar O_2_. (*a*) Pd 3*d* 2D plot of binding energy *versus* temperature; (*b*) Pd 3*d* spectra at selected temperatures (average of three spectra centred at the respective temperature). (*c*) O 1*s* 2D plot binding energy *versus* temperature. (*d*) O 1*s*/Pd 3*d*_3/2_ spectra at selected temperatures (average of five spectra centred at the respective temperature). Photon energy = 750 eV; exit slit = 0.05 mm; pass energy = 10 eV.

The time scale at which spectra with reasonable signal-to-noise ratio can be recorded is of the order of tens of seconds. This is demonstrated by the temperature-programmed measurements shown in Fig. 13 ▸. The spectra show the oxidation of Pd foil in an atmosphere of 2.3 mbar oxygen. Fast O 1*s*/Pd 3*p* and Pd 3*d* spectra were recorded continuously in scanning mode with a photon energy of 750 eV while the sample was annealed at a rate of 3.9 K s^−1^. The time to complete a cycle of O 1*s* and Pd 3*d* spectra was 76 s (32/44 s per spectrum), equivalent to a temperature rise of 5 K. The binding energy *versus* temperature maps in Figs. 13 ▸(*a*) and 13(*c*) illustrate the change in spectra; panels Figs. 13(*b*) and 13(*d*) ▸ display individual spectra at selected temperatures.

Fig. 13 ▸(*a*) clearly shows a transition at 330°C from metallic Pd to PdO through a binding energy shift of the main Pd 3*d*_5/2_ signal from 335.15 eV to 336.75 eV. The change in the Pd signal is accompanied by the growth of the O 1*s* peak at 529.9 eV. Note that the oxygen-related signal in Figs. 13 ▸(*c*) and 13(*d*) is around binding energy 529.8 eV (bulk/surface) and 537.5/538.5 eV (O_2_ gas); the main peak at 531.8 eV is the Pd 3*p*_3/2_ peak. Small O 1*s* and Pd 3*d* oxide signals, due to a thin layer of 2D surface oxide (Lundgren *et al.*, 2002 ▸; Zemlyanov *et al.*, 2006 ▸; Gabasch *et al.*, 2006 ▸), are already observed at the start of the temperature ramp, alongside a dominant peak from metallic Pd in the Pd 3*d* spectrum. The significant reduction of the metallic signal around 330°C indicates the formation of a thick oxide layer at this temperature. This behaviour is in line with ambient-pressure oxidation experiments of Pd{111} by Gabasch *et al.* (2006 ▸), who observed the transition to bulk oxide around 655 K (382°C) under somewhat different experimental conditions. Most of the change in our experiments happens in the temperature range between 300°C and 360°C, over a time period of 15 min, which is clearly on a time scale that can comfortably be observed with the current setup.

Depending on element concentrations and photo-excitation cross sections, faster repetition rates up to around 10 s can be achieved in scanning mode with reasonable signal-to-noise ratios. Thus processes which happen on a minute time scale, such as large-scale surface reconstructions, can be monitored. The snapshot mode of the analyser allows a time resolution of 1 s; however, the width of the spectra is limited to about 12% of the pass energy in this mode (typically <6 eV), which is too small for most applications.

### 5.5. Solid state NEXAFS   

**Figure 14:**
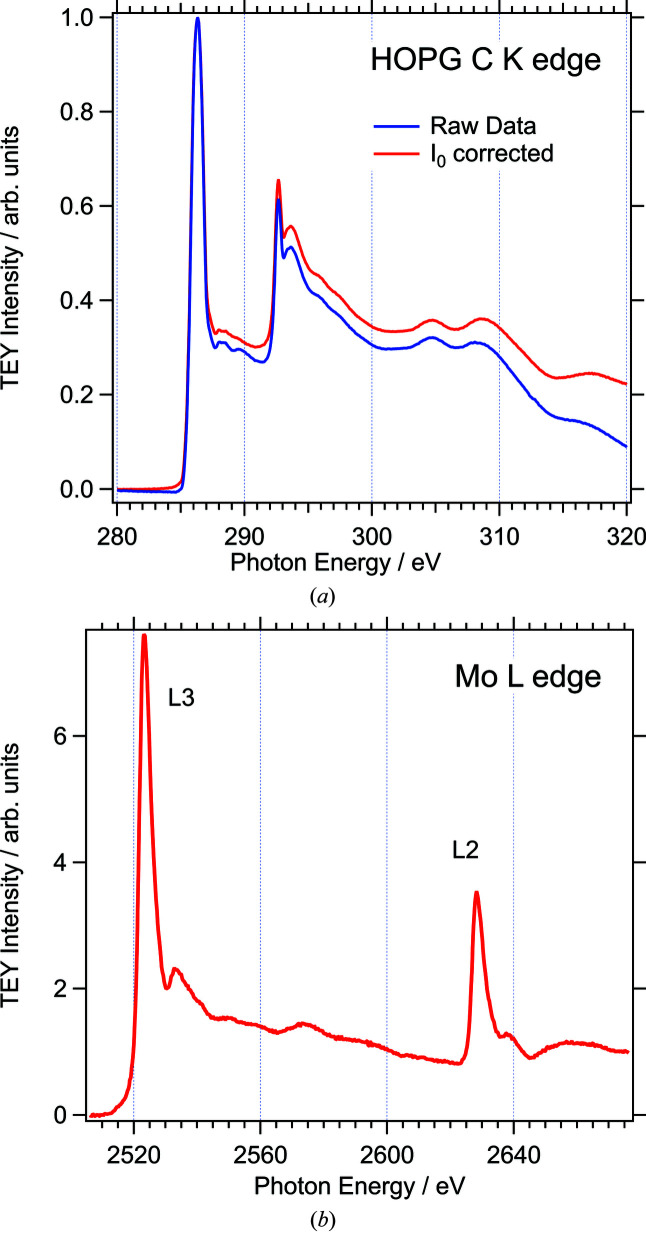
NEXAFS spectra: (*a*) C *K*-edge of highly oriented pyrolitic graphite (HOPG) (grating: 600 lines mm^−1^, exit slit: 0.100 mm). (*b*) Mo *L*_2,3_-edges of molybdenum foil (grating: 1200 lines mm^−1^, exit slit: 0.012 mm).

The NEXAFS spectra in Fig. 14 ▸ demonstrate the capabilities of X-ray absorption spectroscopy at both ends of the photon energy range. Both spectra were measured in vacuum at an angle of incidence of 60°, using the sample current as the TEY signal. Fig. 14 ▸(*a*) shows carbon *K*-edge NEXAFS data of a highly oriented pyrolitic graphite (HOPG) sample. The sample was cleaved in air before being introduced into the analysis chamber. The monochromator was operated with the 600 lines mm^−1^ grating and an exit slit opening of 0.100 mm. The blue curve represents the raw data. In order to remove absorption features from carbon contamination of the beamline, these data were divided by the normalized *I*_0_ spectrum (see Fig. 5 ▸), which leads to the red curve in Fig. 14 ▸(*a*). This procedure removes small artificial features between 288 eV and 291 eV but does not change the overall appearance of the spectrum dramatically, as expected because of the low levels of carbon contamination discussed above. The spectrum is in very good agreement with spectra published earlier (Skytt *et al.*, 1994 ▸; Jeong *et al.*, 2008 ▸). The sharp π and exciton resonances at 286.2 eV and 292.6 eV, respectively, show the same or smaller widths than those in earlier publications.

Fig. 14 ▸(*b*) shows the Mo *L*_3_- and *L*_2_-edges in the photon energy range between 2500 eV and 2680 eV. The spectrum was recorded with the 1200 lines mm^−1^ monochromator grating and exit slit opening of 0.012 mm. The sample was a molybdenum foil, which had been polished and cleaned in acetone before being introduced into the analysis chamber. It is therefore expected to be oxidized at the surface. The excitation energies reported in the literature for Mo *L*_3_-edges of different oxidation states vary between around 2520 eV and 2530 eV (Evans & Mosselmans, 1991 ▸; Aritani *et al.*, 2001 ▸; George *et al.*, 2009 ▸; Wawro *et al.*, 2018 ▸). For the data shown here, the energy axis was calibrated in line with the value reported by Wawro *et al.* (2018 ▸) for metallic Mo, 2523 eV. The spectrum is in good qualitative agreement with metallic Mo *L*-edge spectra published earlier (Evans & Mosselmans, 1991 ▸; Wawro *et al.*, 2018 ▸). The widths of the two main peaks (FWHM = 5.0/4.3 eV) are well within the range of those in spectra of Mo metal and inorganic Mo compounds reported in the literature.

## 6. Summary and outlook   

The ambient-pressure endstation and beamline branch at the VerSoX beamline (B07) of Diamond Light Source provide a versatile facility for XPS and NEXAFS measurements in the mbar pressure range. The beamline has a maximum resolving power *h*ν/Δ(*h*ν) > 5000 with a photon flux > 10^10^ photons s^−1^ from 170 eV to 2000 eV and can be operated (delivering lower flux) up to 2800 eV. Operating the beamline in an oxygen atmosphere of 10^−8^ mbar eliminates carbon contamination of the optical elements almost completely. The endstation is equipped with a differentially pumped electron energy analyser and beamline entrance allowing pressures at the sample of up to 100 mbar while maintaining sufficiently low pressures in the beamline and the detector part of the analyser. XPS data can be measured routinely up to 30 mbar with sufficient signal. Beamline entrance and analyser are combined in one flange such that sample environments can be exchanged relatively easily without the need of beamline re-alignment. Currently two sample environments are available, a small reaction cell (‘Tea Cup’) and a larger chamber (‘Tea Pot’), which allows *in vacuo* sample transfer from a UHV preparation chamber, sample storage, and/or load lock. The data presented here demonstrate the capability of the instrument to analyse details of the surface composition of solid samples under ambient-pressure conditions using XPS and NEXAFS. They also show that the gas phase can be analysed through X-ray absorption spectroscopy. Short XPS spectra can be measured at a time scale of tens of seconds. The shortest data acquisition times for NEXAFS are around 0.5 s per data point.

The ambient-pressure endstation and beamline are part of a suite of instruments currently under construction at VerSoX. In its final stage, the facility will consist of two completely independent beamline branches, which use the radiation from the same bending magnet. The second branchline will enable NEXAFS measurements at higher pressures, up to 1 bar, and high-throughput XPS under vacuum conditions, thus expanding the accessible pressure range of the instrument described here at both ends.

## Supplementary Material

Supporting Figures S1 to S5. DOI: 10.1107/S1600577520009157/ve5129sup1.pdf

## Figures and Tables

**Table 1:** Details of the optical elements of B07 branch C All mirrors and gratings are based on Si single-crystal substrates. M1c has a 5–10 nm Cr binding layer between substrate and coating; the other optical elements have no binding layers.

Element	Surface coating	Distance from source
Mirror M1c (torroid)	Rh (50 nm)†	13.124 m
Channel-cut crystal monochromator	None (Si)	20.133 m
cPGM mirror M2c (plane)	Pt/Rh (40 nm)	21.334–21.784 m
cPGM grating 400 lines mm^−1^	Au (40 nm)	21.824 m
cPGM grating 600 lines mm^−1^ (blazed)	Pt/Au (20/30 nm)	21.824 m
cPGM grating 1200 lines mm^−1^	Au (40 nm)	21.824 m
Mirror M3c (cylinder)	Rh (50 nm)	23.324 m
Mirror M4c (cylinder)	Rh (50 nm)	33.324 m
Mirror M5c (ellipse)	Rh (50 nm)	35.824 m

† 5–10 nm Cr binding layer between Si substrate and Rh coating.
