# Supplementary material for: Ambient-pressure endstation of the Versatile Soft X-ray (VerSoX) beamline at Diamond Light Source
Source: J Synchrotron Radiat. 2020 Aug 17;27(Pt 5):1153–66. doi: 10.1107/S1600577520009157 (PMC7467337; doi:10.1107/S1600577520009157)
Supplement: Supplementary file 1 [file s-27-01153-sup1.pdf]

JOURNAL OF SYNCHROTRON RADIATION

Volume 27 (2020)

Supporting information for article:

Ambient-pressure endstation of the Versatile Soft X-ray (VerSoX) beamline at Diamond Light Source

Georg Held, Federica Venturini, David Grinter, Pilar Ferrer, Rosa Arrigo, Liam Deacon, Wilson Quevedo Garzon, Kanak Roy, Alex Large, Christopher Stephens, Andrew Watts, Paul Larkin, Matthew Hand, Hongchang Wang, Linda Pratt, James J. Mudd, Thomas Richardson, Suren Patel, Michael Hillman and Stewart Scott

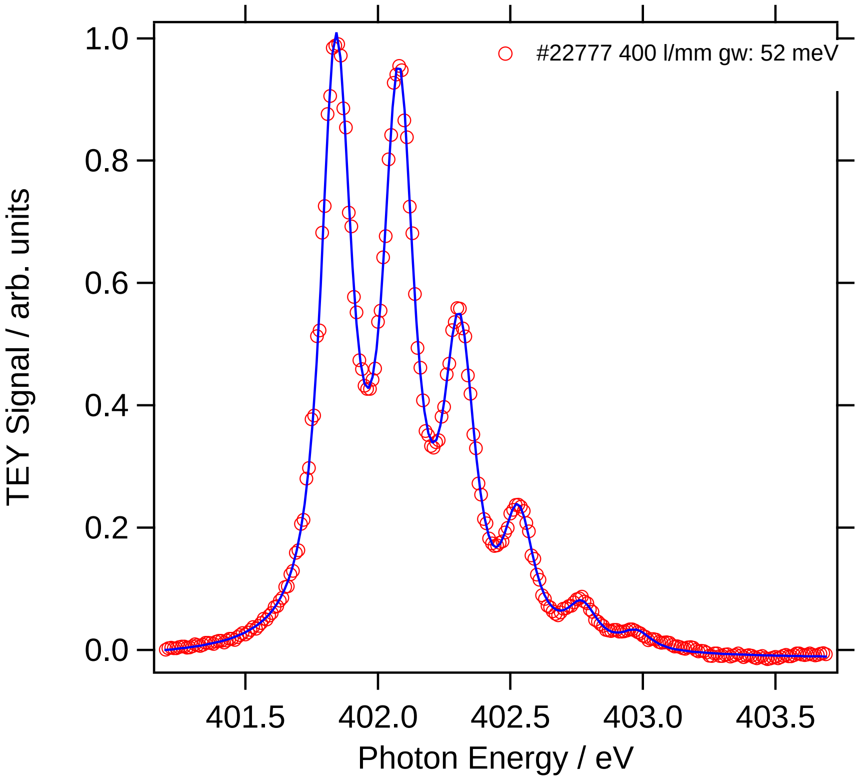

Figure S1: N_2 K-edge absorption spectrum demonstrating the maximum resolving power for the 400 l mm^{-1} grating and exit slit 0.0125 mm. The spectrum was fitted with a sum of Voigt functions with fixed Lorentzian width of 115 meV, which leads to a Gaussian width of 52 meV, i.e. a resolving power $h\nu/\Delta(h\nu)$ of 7692.

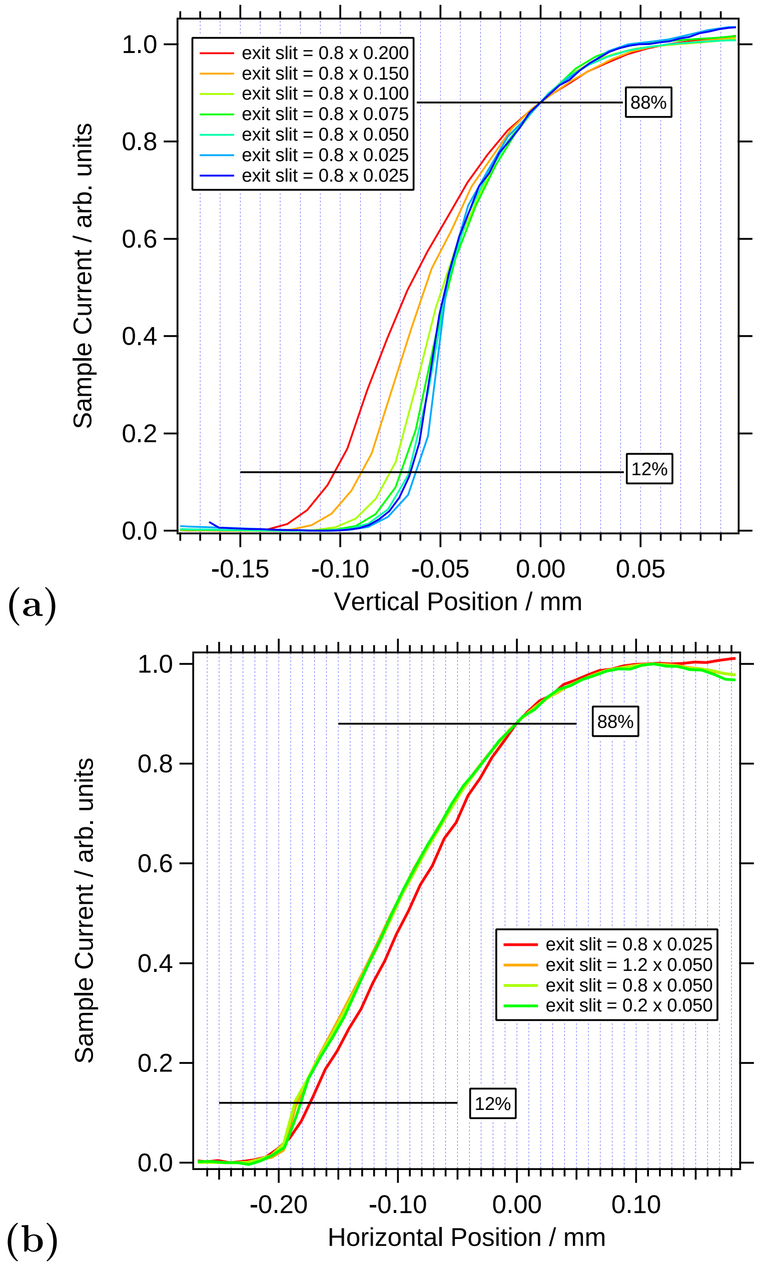

Figure S2: Scans across a mica edge in (a) vertical and (b) horizontal direction while measuring the sample current for different exit slit openings (horizontal × vertical gap size). The beam size is determined as the distance between the 12% and 88% level. Note, the angle of incidence was 30° in these measurements, therefore the footprint in the horizontal direction is 2 ($= (\sin 30°)^{-1}$) times the beam size.

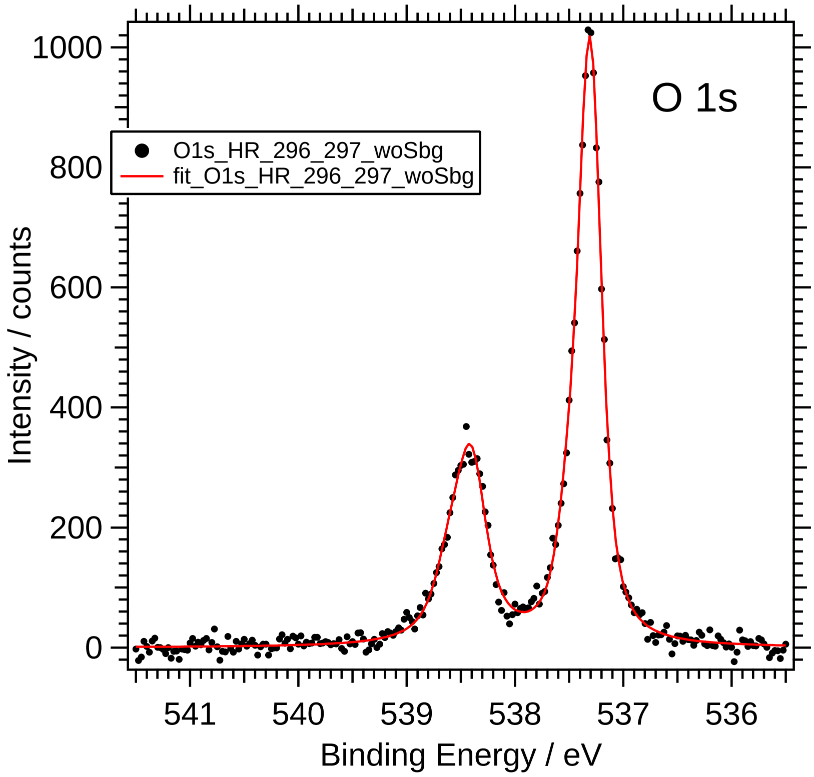

Figure S3: O 1s XPS spectrum for gas-phase O_2 (gas pressure: 1.0 mbar; photon energy: 750 eV; pass energy: 2.5 eV; grating: 600 l mm^{-1}; exit slit: 0.012 mm). The FWHM of the low BE peak is 301 meV, which corresponds to instrumental broadening of 210 meV (fitted with a Voigt function of 149 meV lifetime broadening).

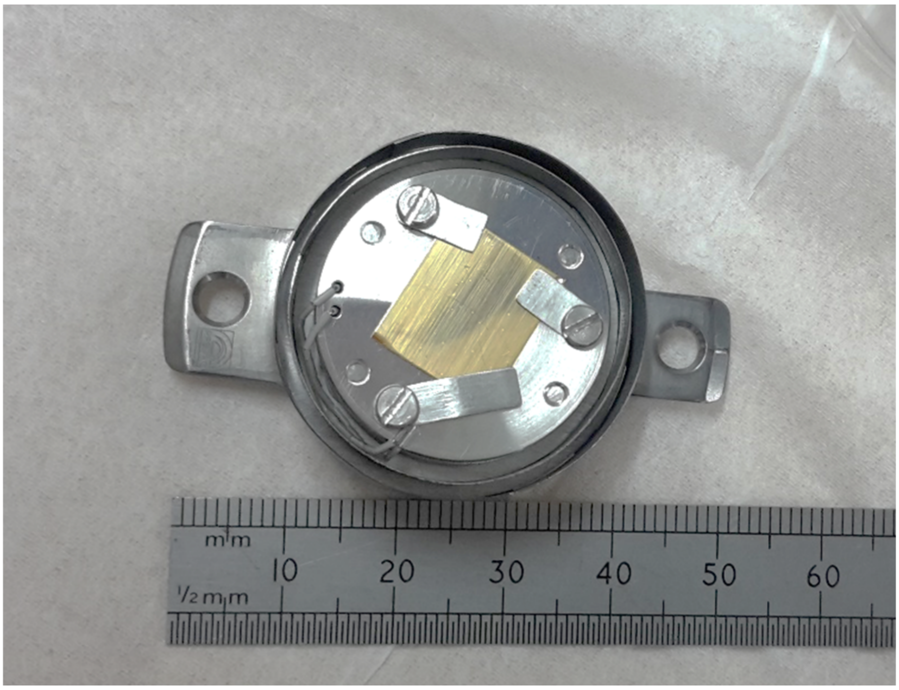

Figure S4: Ambient-pressure sample holder for the Tea Pot manipulator. The diameter of the central sample plate is 25 mm. The thermocouple wires can be seen at the bottom left. This is one example of Prevac "PTS"-style sample holders that can be used with the Tea Pot manipulator. A full list of sample holder options can be found on the Prevac website (https://www.prevac.eu/).

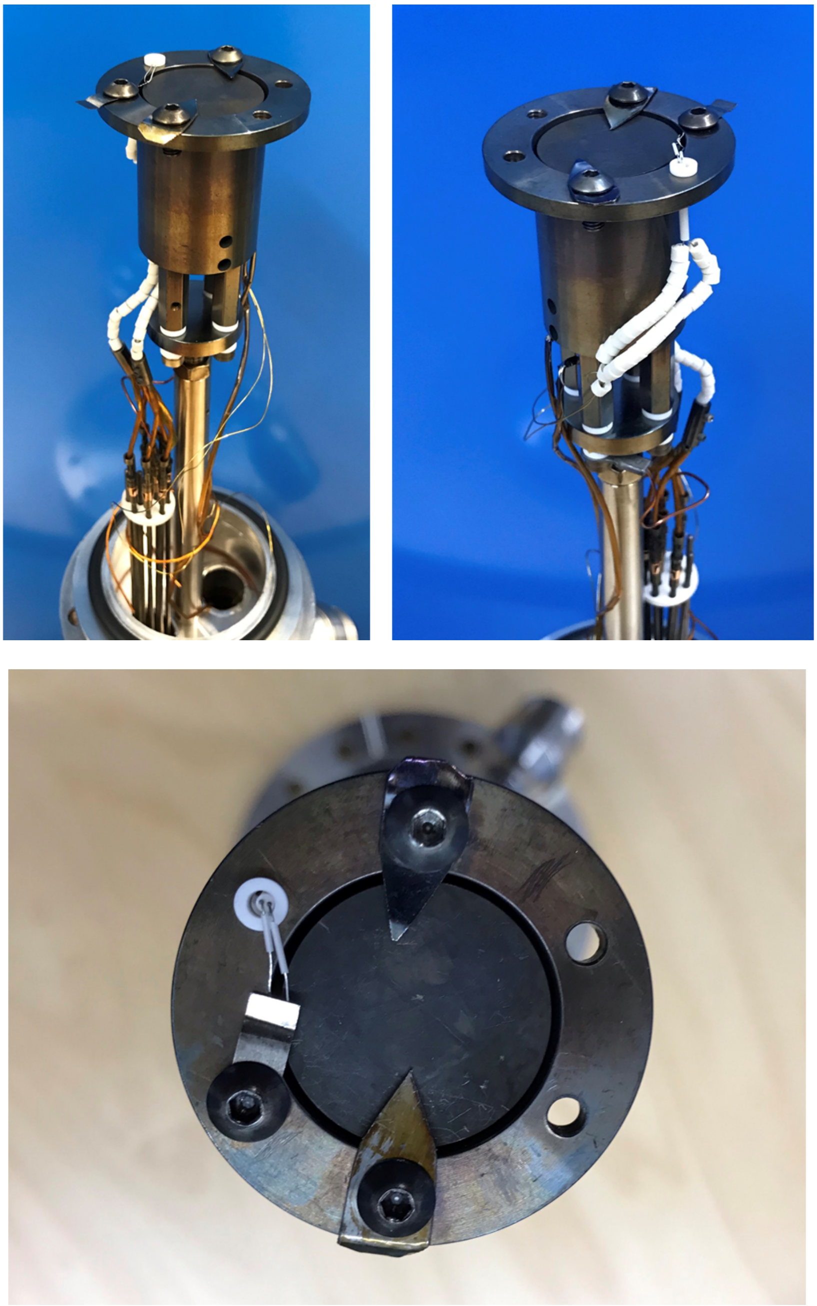

Figure S5: Sample holder and heater for the Tea Cup. Top: side views from different angles; bottom: top view. The diameter of the button heater in the center is 25 mm. The thermocouple wires can be seen on the left in the top-view image.
